# Influence of parent vessel feature on the risk of internal carotid artery aneurysm rupture via computational method

**DOI:** 10.1038/s41598-023-47927-7

**Published:** 2023-11-23

**Authors:** Mehdi Fattahi, Seyyed Amirreza Abdollahi, Ali Hosin Alibak, Saleh Hosseini, Phuyen Dang

**Affiliations:** 1 Institute of Research and Development, Duy Tan University, Da Nang, Vietnam; 2 School of Engineering and Technology, Duy Tan University, Da Nang, Vietnam; 3 Faculty of Mechanical Engineering, University of Tabriz, Tabriz, Iran; 4 Petroleum Engineering Department, Faculty of Engineering, Soran University, Soran, Kurdistan Region 44008 Iraq; 5 Department of Chemical Engineering, University of Larestan, Larestan, Iran

**Keywords:** Biomedical engineering, Mechanical engineering

## Abstract

In this study, the role of sac section area and parent vessel diameter on the hemodynamic feature of the blood flow in selected internal carotid artery (ICA) aneurysms is comprehensively investigated. The changes of wall shear stress, pressure, and oscillatory shear index (OSI) of blood stream on the vessel for various aneurysms with coiling treatment. To attain hemodynamic factors, computational technique is used for the modeling of non-Newtonian transient blood flow inside the three different ICA aneurysms. Three different saccular models with various Parent vessel mean Diameter is investigated in this study. The achieved outcomes show that increasing the diameter of the parent vessel directly decreases the OSI value on the sac surface. In addition, the mean wall shear stress decreases with the increase of the parent vessel diameter.

## Introduction

Intracranial aneurysms are abnormal bulges or weak areas in the walls of blood vessels within the brain, particularly in the internal carotid artery (ICA). These aneurysms pose a significant risk of rupture, leading to potentially life-threatening hemorrhages. The rupture of an intracranial aneurysm remains a critical challenge in neurosurgery, and understanding the underlying hemodynamic factors contributing to aneurysm rupture is crucial for developing effective treatment strategies^1–3^.

Hemodynamics, which refers to the study of blood flow patterns and forces within blood vessels, plays a vital role in the initiation, growth, and rupture of intracranial aneurysms. Various hemodynamic factors, such as wall shear stress, flow velocity, and pressure distribution, influence the structural integrity of an aneurysm. For instance, elevated wall shear stress and flow impingement on the aneurysm wall can lead to progressive weakening and eventual rupture^4,5^. There are several papers related to biomedical study via theoretical techniques^6–11^. The focused on different aspects of biomedical science^12–17^.

To mitigate the risk of rupture, endovascular techniques, such as coiling, have emerged as an effective treatment option^18–20^. Coiling involves the placement of a small metal coil into the aneurysm sac to promote thrombosis and reduce blood flow into the aneurysm. However, the precise hemodynamic alterations induced by coiling and their impact on the risk of aneurysm rupture are not yet fully understood. There are various treatments for the problem related to human’s health^21–27^

Computational fluid dynamics (CFD) has emerged as a valuable tool for investigating the hemodynamics of intracranial aneurysms and evaluating the effectiveness of treatment strategies. By simulating blood flow within patient-specific vascular geometries, CFD allows researchers to quantify key hemodynamic parameters and analyze their relationship with aneurysm rupture risk. This approach provides valuable insights into the complex flow patterns, wall shear stress distribution, and pressure gradients within the aneurysm^28–30^.

The findings of this study have the potential to enhance our knowledge of the hemodynamic mechanisms underlying aneurysm rupture and the efficacy of coiling as a treatment strategy. Ultimately, this research may contribute to the development of improved clinical decision-making tools and personalized treatment approaches for patients with ICA aneurysms, with the goal of minimizing the risk of rupture and optimizing patient outcomes^31^.

Several key hemodynamic parameters are commonly studied in relation to aneurysm rupture risk^32,33^. These parameters provide insights into the flow patterns and forces acting on the aneurysm wall, which can play a crucial role in aneurysm initiation, growth, and rupture. Some of the key hemodynamic parameters will be explained. Wall Shear Stress (WSS) is the tangential force exerted by flowing blood on the vessel wall. Elevated WSS has been associated with increased aneurysm rupture risk. High WSS can lead to endothelial dysfunction, inflammation, and remodeling of the vessel wall, potentially weakening it over time. Flow velocity refers to the speed at which blood flows through the aneurysm and adjacent vessels. Disturbed flow patterns, characterized by high-velocity jets, vortices, and flow impingement on the aneurysm wall, have been linked to an increased risk of aneurysm rupture. Pressure distribution within the aneurysm sac and surrounding vessels can influence the mechanical stress on the aneurysm wall. High-pressure gradients and localized pressure peaks within the aneurysm may contribute to wall degeneration and rupture^34,35^.

The complex flow patterns within the aneurysm, such as recirculation zones and flow impingement, are of interest in understanding the hemodynamics of aneurysms. Disturbed flow patterns can result in regions of low wall shear stress or high pressure, which may promote aneurysm growth and rupture. Energy loss quantifies the dissipation of kinetic energy within the aneurysm^36^. Higher energy loss has been associated with increased rupture risk, as it indicates inefficient flow patterns and flow disturbances that can weaken the aneurysm wall. Oscillatory Shear Index (OSI) measures the degree of oscillatory flow and changes in the direction of shear stress. High OSI values have been correlated with aneurysm initiation and progression, suggesting a potential role in rupture risk assessment. By studying these and other hemodynamic parameters, researchers can gain valuable insights into the complex interplay between blood flow dynamics and aneurysm rupture. Computational fluid dynamics (CFD) simulations and advanced imaging techniques play a crucial role in quantifying these parameters and investigating their relationship with aneurysm behavior, providing a foundation for improved risk prediction and treatment strategies.

In this study, we aim to explore the role of hemodynamic factors on the risk of rupture in ICA aneurysms and investigate how the coiling technique influences these factors using a computational fluid dynamics approach. By integrating patient-specific vascular geometries, physiological flow conditions, and accurate modeling of coiling procedures, we can gain a deeper understanding of the hemodynamic modifications induced by coiling and their implications for aneurysm rupture risk reduction.

Although several investigations have analyzed the aneurysm feature on the risk of bleeding, limited research has presented valuable information on the role of parent vessel mean diameter on the hemodynamic characteristics of blood flow inside the ICA aneurysms. This work tries to investigate the role of Parent vessel mean diameter on the hemodynamic factors related to the bleeding of the saccular ICA aneurysms. Computational fluid dynamic is used to visualize the blood stream inside real three-dimensional ICA aneurysms. Wall shear stress and OSI value in various stages of the cardiac cycle.

## Governing equations and numerical method

For the selection of the ICA aneurysms, more than 100 aneurysms with different geometrical sizes are obtained from Aneurisk^37^ and their geometrical features are compared based on the sac section area and parent mean diameter. It is confirming that all methods were carried out in accordance with relevant guidelines and regulations. Besides, all experimental protocols were approved by of the Ca’ Granda Niguarda Hospital and it is confirmed that informed consent was obtained from all subjects and/or their legal guardian(s).

**Figure 1:**
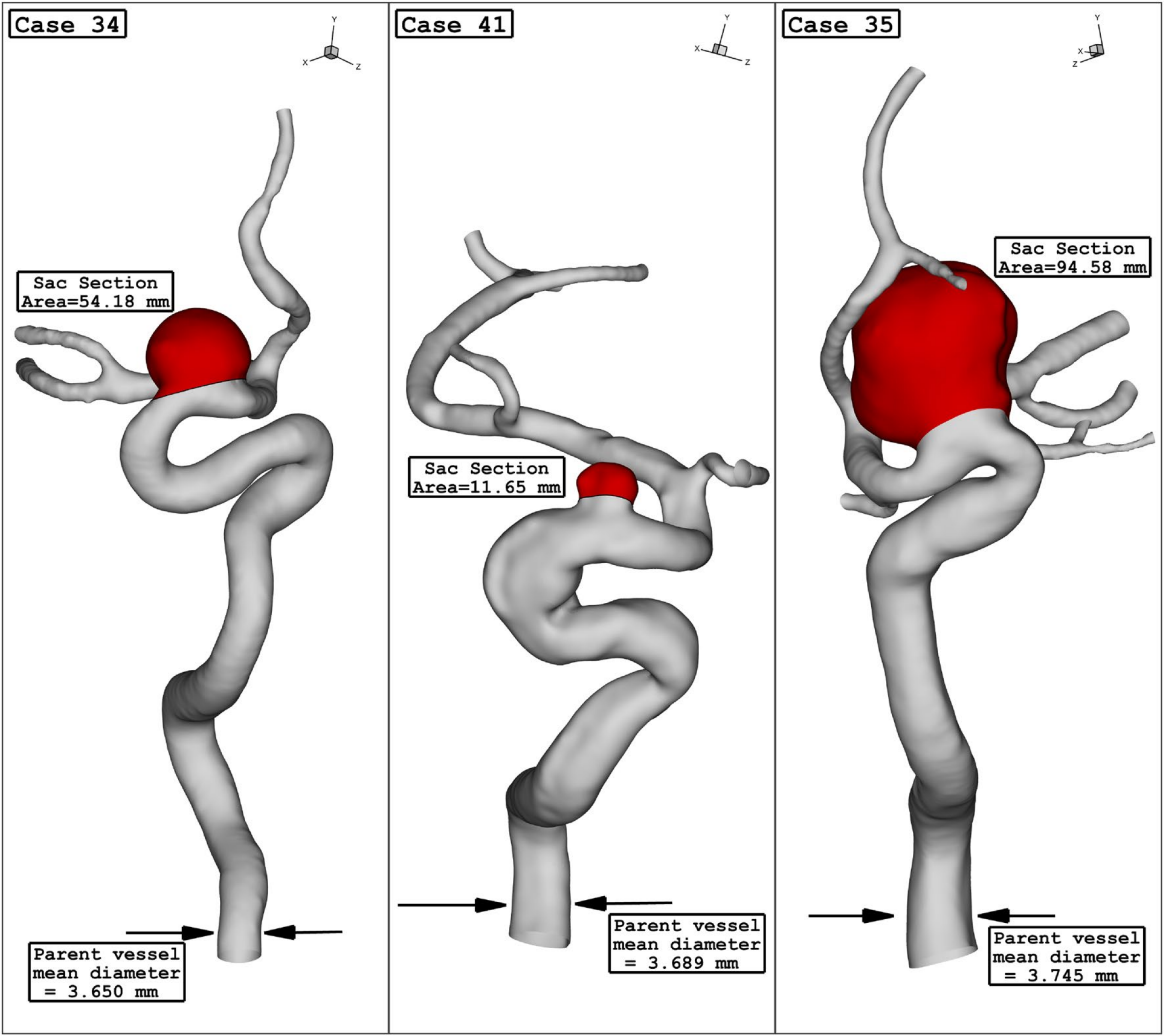
ICA aneurysm geometry of 3 different cases.

Among these cases, three cases are chosen (Fig. 1), and details of the selected aneurysms are presented in Table 1. As presented in the table, three female patients with different sizes of sac section area and parent vessel mean diameter. The HCT of the blood is 0.4 since the patients are female. Since the aneurysms are filled with coiling, it is assumed that the porous media is applied in the saccular domain. In Table 2, the facts of applied porosity and viscous residence (permeability) are presented.

**Table 1:** geometrical details of chosen ICA sac.

Case ID	Parent vessel mean diameter (mm)	Sac section area (mm^2^)	Sex
34	3.650	54.18	Female (HCT = 0.40)
41	3.689	11.65	Female (HCT = 0.40)
35	3.745	94.58	Female (HCT = 0.40)

**Table 2:** Coiling characteristic inside aneurysm sac.

HCT	Porosity	Viscous resistance (m^2^/l)
0.4	0.844	16,958,264.02

The study of the blood stream moves inside the parent vessel and saccular aneurysm requires specific considerations. Transient Navier Stokes equations are used for the modeling of the blood flow inside the aneurysms^38–40^. One-way FSI approach is considered for the interaction of the vessel and aneurysm wall with blood flow^30,41^. Due to non-Newtonian blood characteristics, the Casson model is conventionally applied to calculate the viscosity of the blood within the vessel^32,42^. The flow is assumed laminar since the velocity of the blood is not high. ANSYS-FLUENT is used for the modeling of the blood flow within saccular aneurysms^43^. This software used for different engineering applications^44–49^

**Figure 2:**
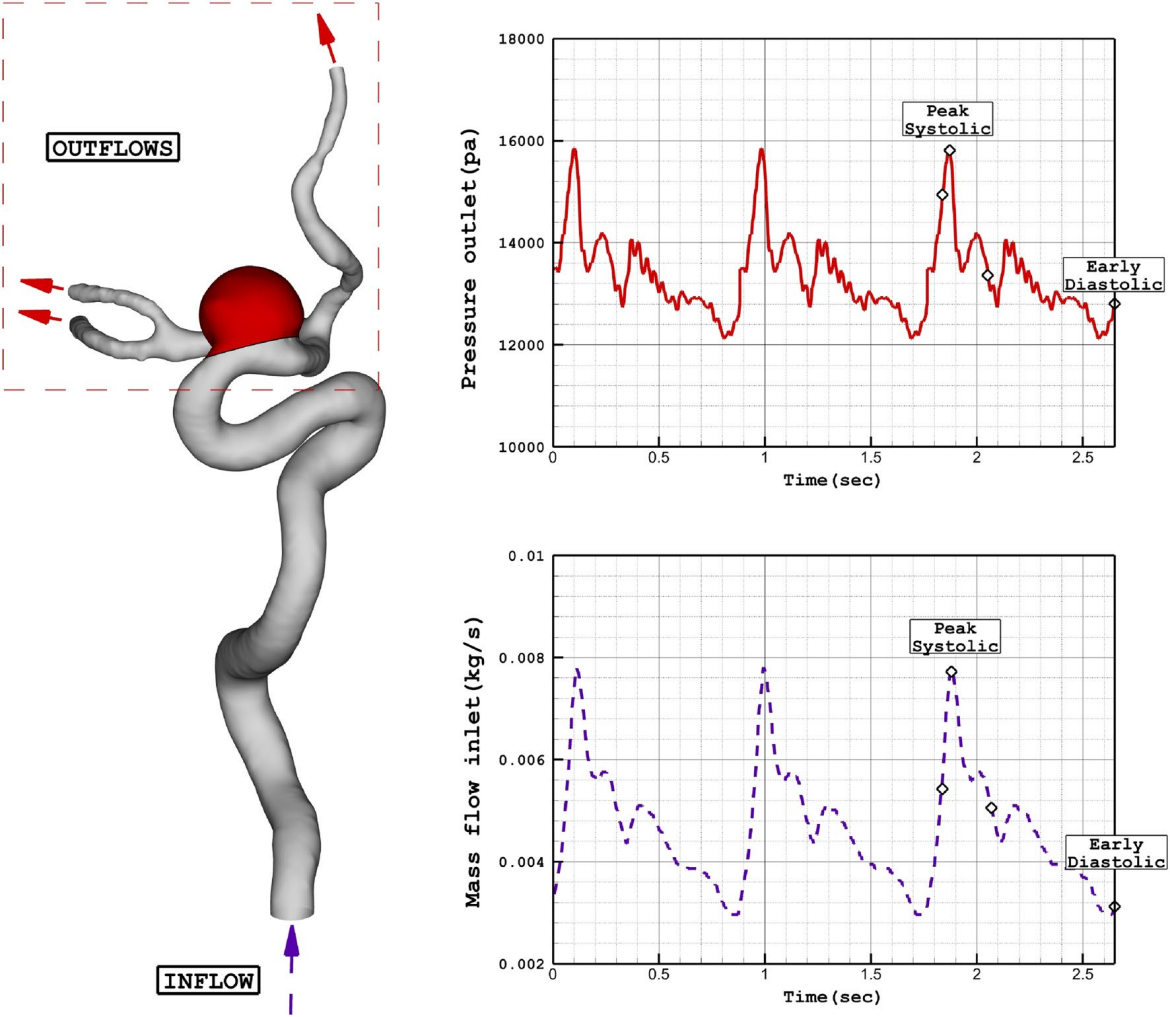
Applied mass and pressure profile at inlet and outlets.

**Figure 3:**
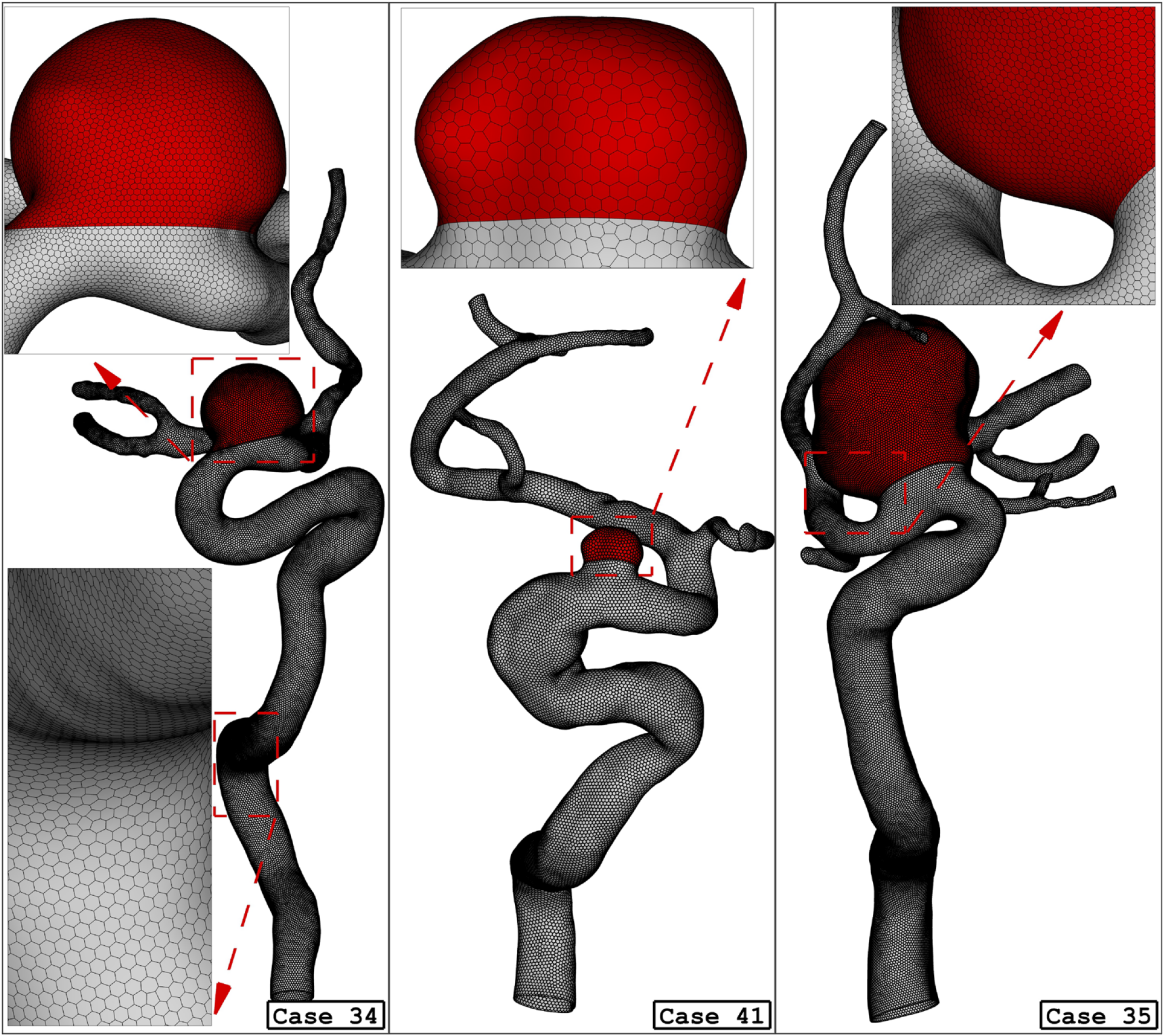
Grid generation for 3 different ICA cases.

Since the blood flow inside the aneurysms and parent vessel is transient, mass flow rate and pressure outlet are applied at the inlet and outlet, respectively. Figure 2 plots the applied mass and pressure profile at the inlet and outlet, respectively. The maximum flow rate of the blood is at peak systolic and the end of the cardiac cycle is named early diastolic. In the former, wall shear stress is reported while the OSI index is reported at the end of the cardiac cycle. Figure 3 illustrates the produced grid for the chosen cases with a close-up view. As shown in Fig. 3, the hexagonal uniform structured grids are used for the selected saccular and parent vessels. The number of produced grids for these models is between 840,000 and 1,100,000 cells.

## Results and discussion

The hemodynamic results of the simulation of the blood inside the selected aneurysm are presented in Table 3. The mean and minimum WSS, mean OSI, mean wall pressure, and mean blood velocity inside the aneurysm are presented in Table 3. Full contour and plots are presented subsequently to explain the main effective terms on the hemodynamic factors.

**Table 3:** Details of hemodynamic analyses.

	Parent vessel mean diameter (mm)	Sac section area (mm^2^)	WSS_mean (Pa)	WSS_min (Pa)	OSI_mean	Wall pressure_mean (Pa)	Aneurysm velocity_mean (m/s)
Case 34	3.650	54.18	8.146665	0.130785	0.02603191	25,071.71	0.4034861
Case 41	3.689	11.65	4.200369	0.155615	0.01506075	24,503.94	0.1524694
Case 35	3.745	94.58	2.143452	0.075405	5.64e−5	20,665.31	0.1365955

**Figure 4:**
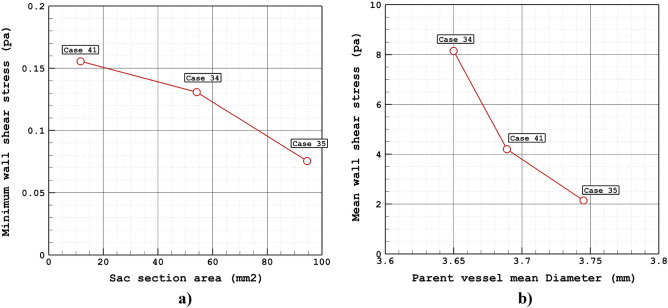
(**a**) Minimum, (**b**) mean wall shear stress vs sac section are at peak systolic.

**Figure 5:**
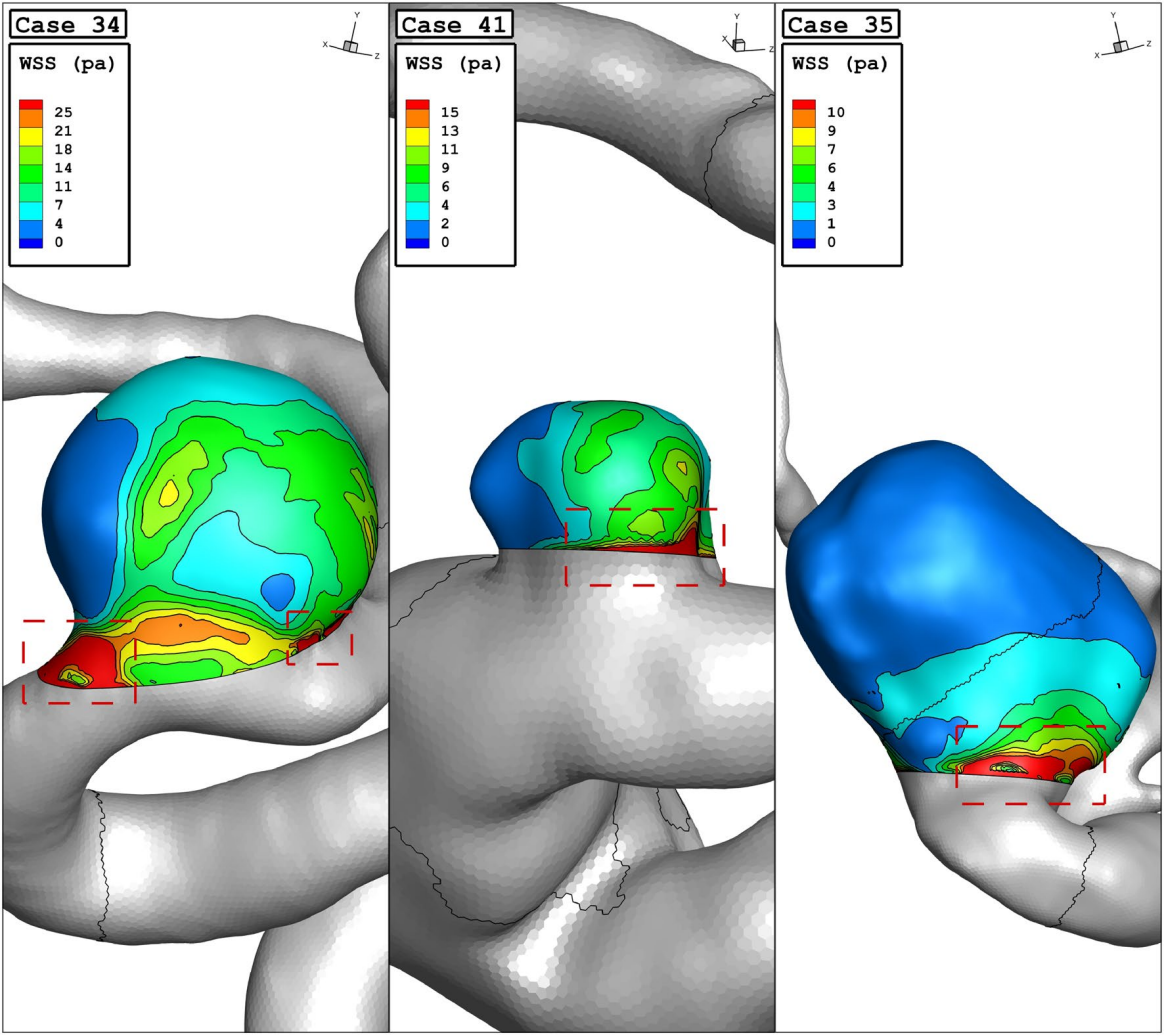
WSS contours (peak systolic) in different cases.

Figure 4a plots the variation of the minimum wall shear stress of selected aneurysms in different sac section areas. The archived results indicate that the minimum wall shear stress is decreased by about 50% by increasing of the sac section area from 11 to 94 mm^2^. Considerable reduction in this change may related to the high increase of incoming blood flow by increasing of sac section area. In Fig. 4b, the effects of vessel diameter on the mean wall shear stress are disclosed. As the mean diameter of the parent vessel increases slightly, the value of mean wall shear stress drops from 8 pas to 2 Pa. Figure 5 illustrates the contour of WSS of different cases at peak systolic. Variation of the WSS indicates that the value of this index is higher near the neck area where incoming blood flow enters the sac and returns to the parent vessel. It is also perceived that the WSS in the dome section is not high.

**Figure 6:**
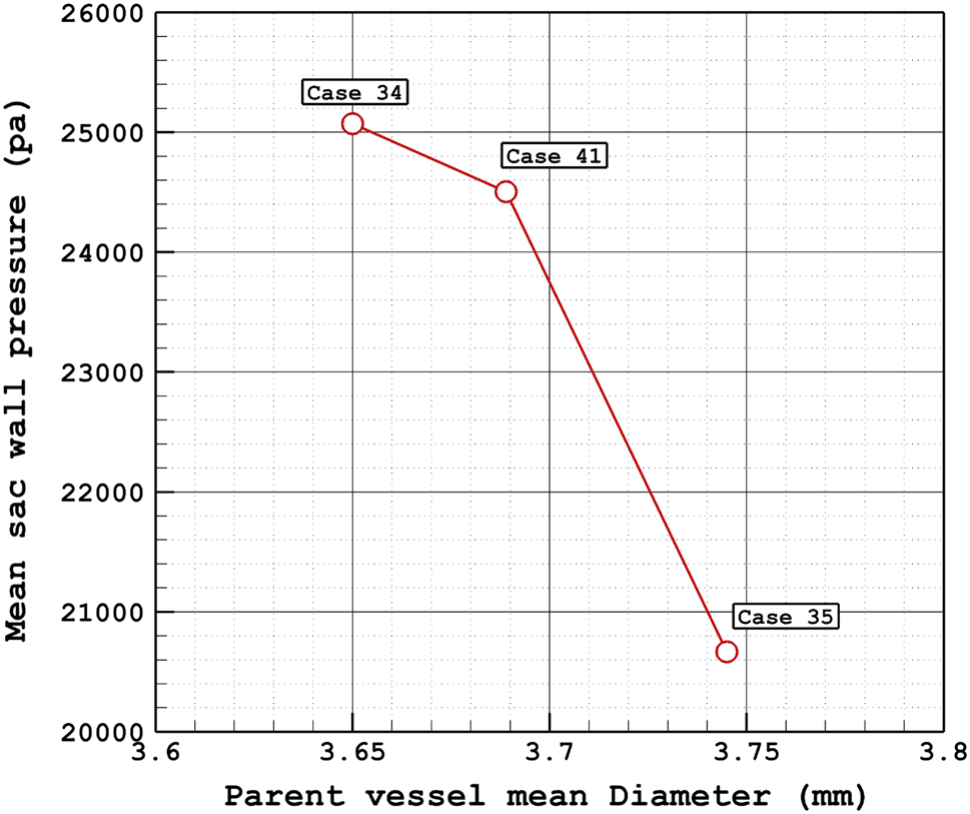
Mean wall pressure vs parent vessel mean diameter at peak systolic.

**Figure 7:**
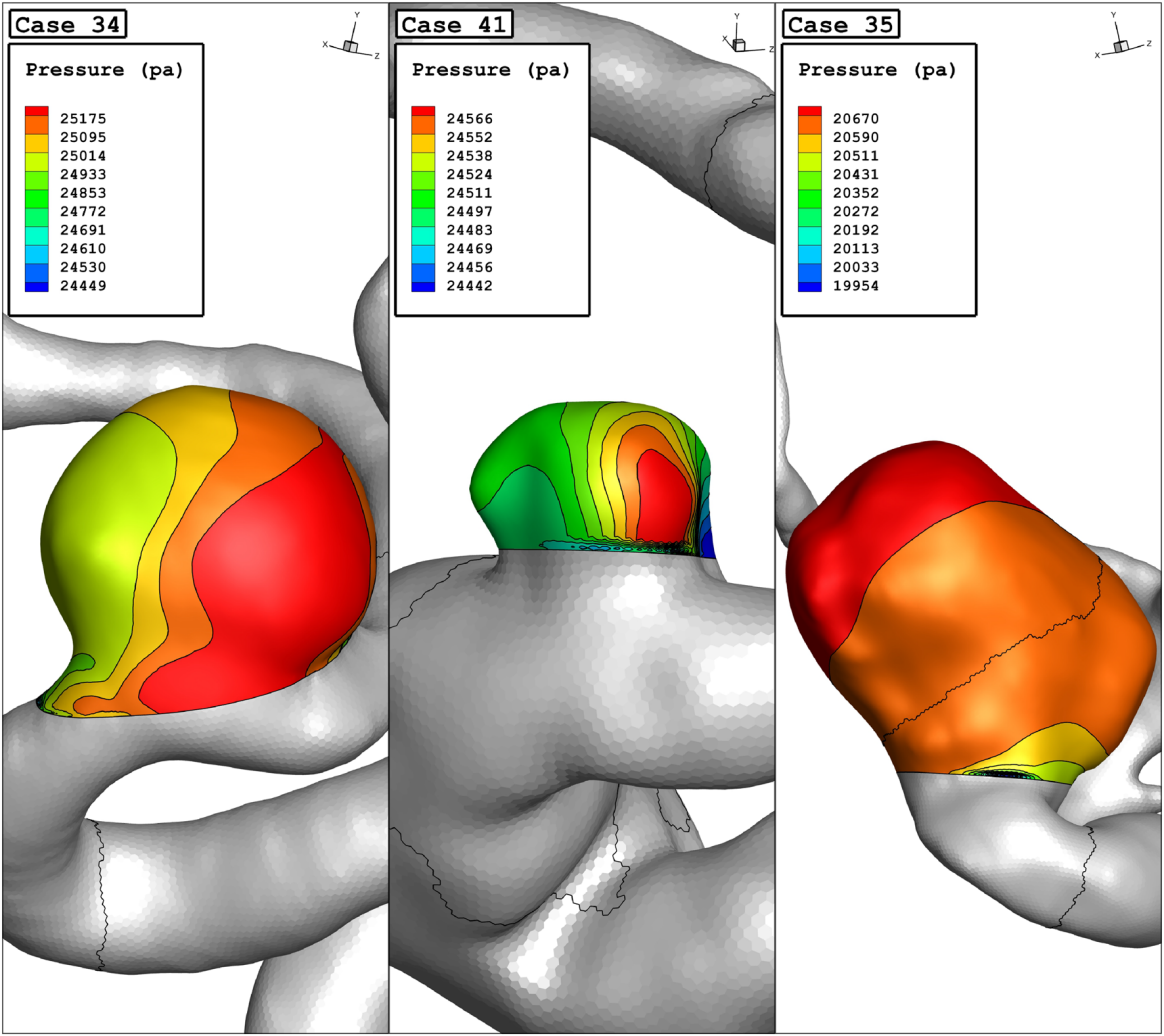
Wall pressure contours (peak systolic) in different cases.

The variation of the mean sac wall pressure of the selected aneurysm in a diverse range of parent vessel means diameter at peak systolic stage is demonstrated in Fig. 6. It is observed that pressure changes happen due to the changes in the parent vessel's mean diameter. As the size of the parent vessel diameter is increased, the mean pressure of the blood flow inside the sac is decreased. Figure 7 illustrates the contour of the pressure on the sac surface at peak systolic. The variation of the pressure shows that the maximum pressure on the sac wall occurs in the region where the incoming blood contact with the wall of the aneurysm.

**Figure 8:**
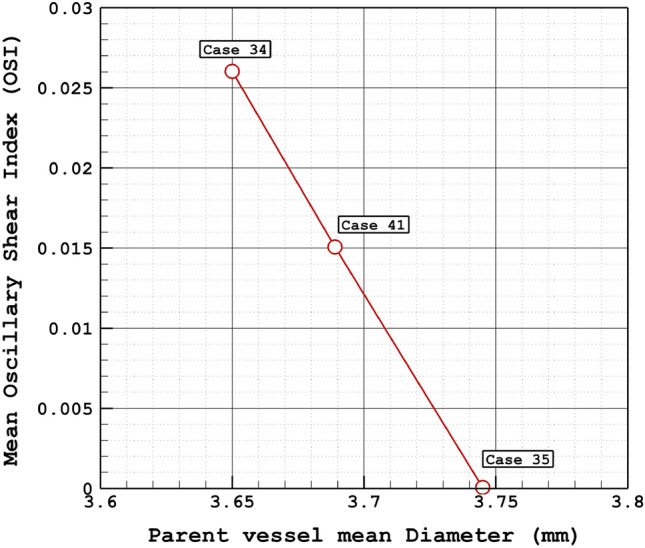
Mean OSI vs parent vessel mean diameter at early diastolic.

**Figure 9:**
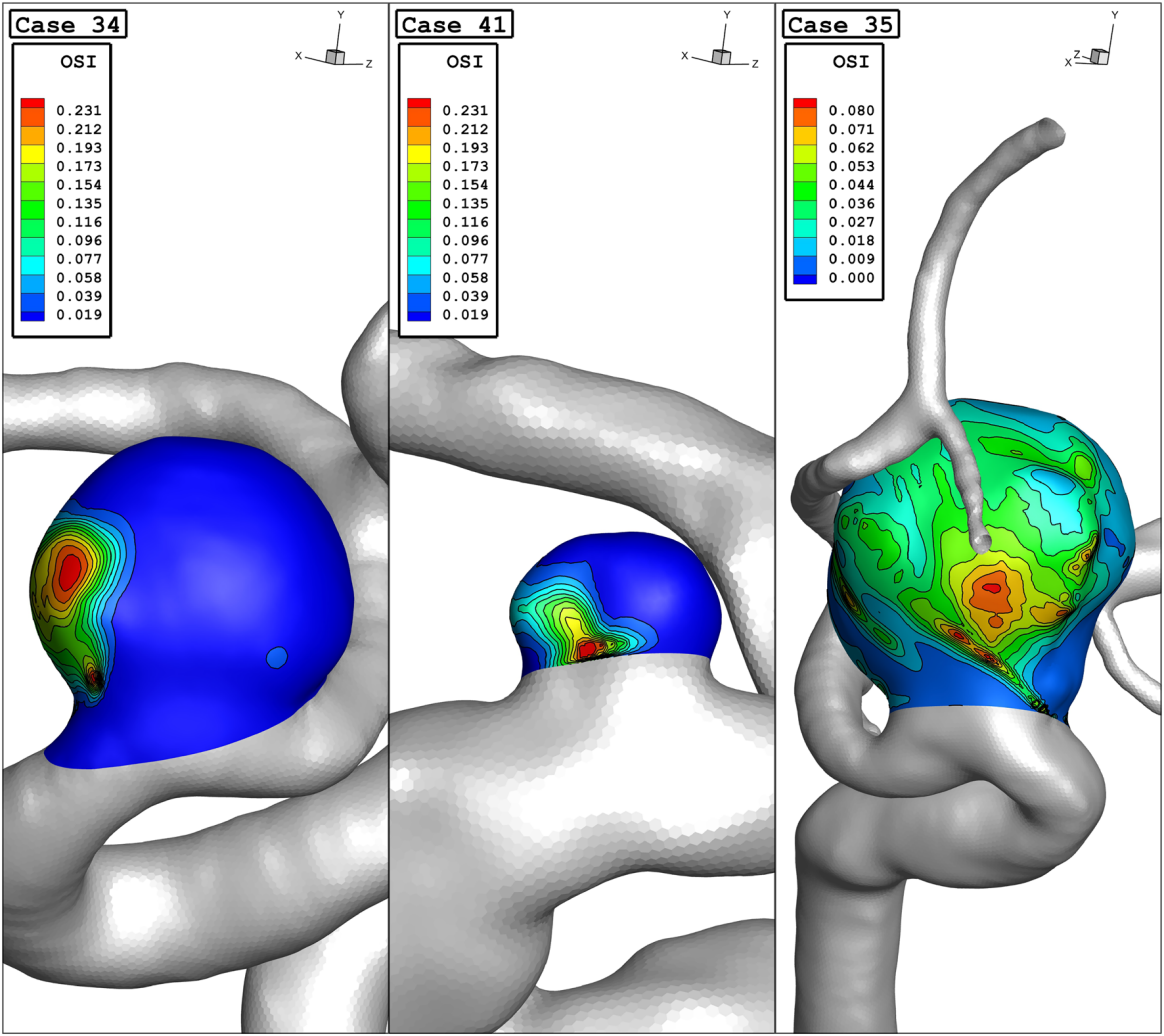
OSI contours (early diastolic) in different cases.

The variation of the mean oscillatory shear index (OSI) at early diastolic for various parent vessel mean diameters is demonstrated in Fig. 8. The OSI value is decreased as the parent vessel diameter is increased. In case 35, the mean value of OSI is approximately zero which means that the changes in OSI in this model are considerably lower than other models. Figure 9 confirms that the range of OSI on the sac surface of case 35 is lower than in the other two cases. Indeed, this model has a larger sac section area than others and its parent vessel diameter is also more than the other two cases.

**Figure 10:**
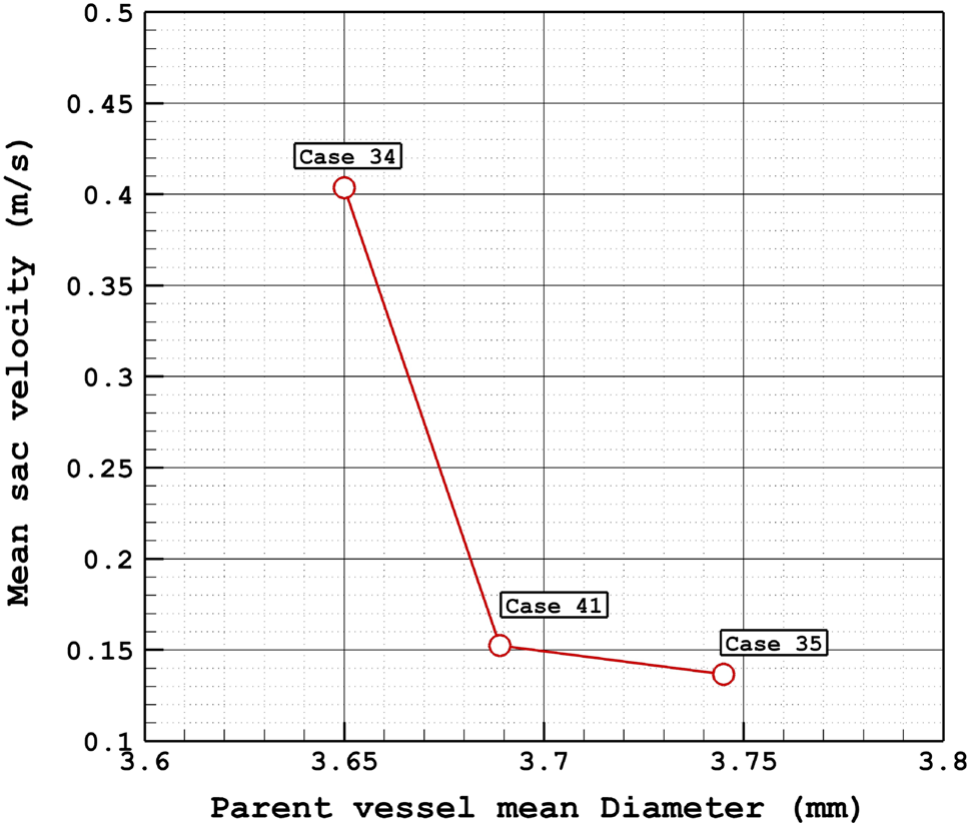
Mean sac velocity vs parent vessel mean diameter at peak systolic.

**Figure 11:**
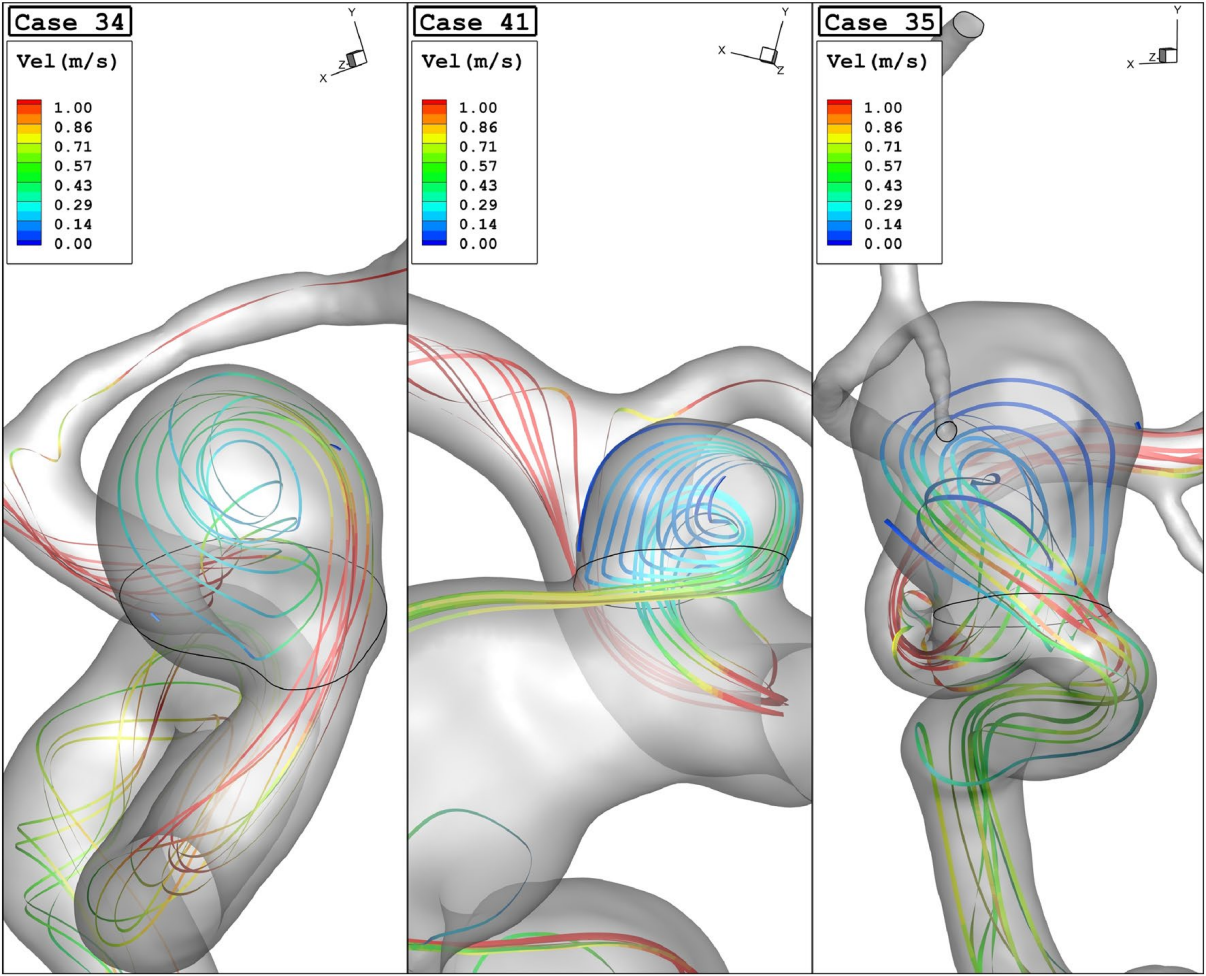
Streamlines (velocity at peak systolic) in different cases.

**Figure 12:**
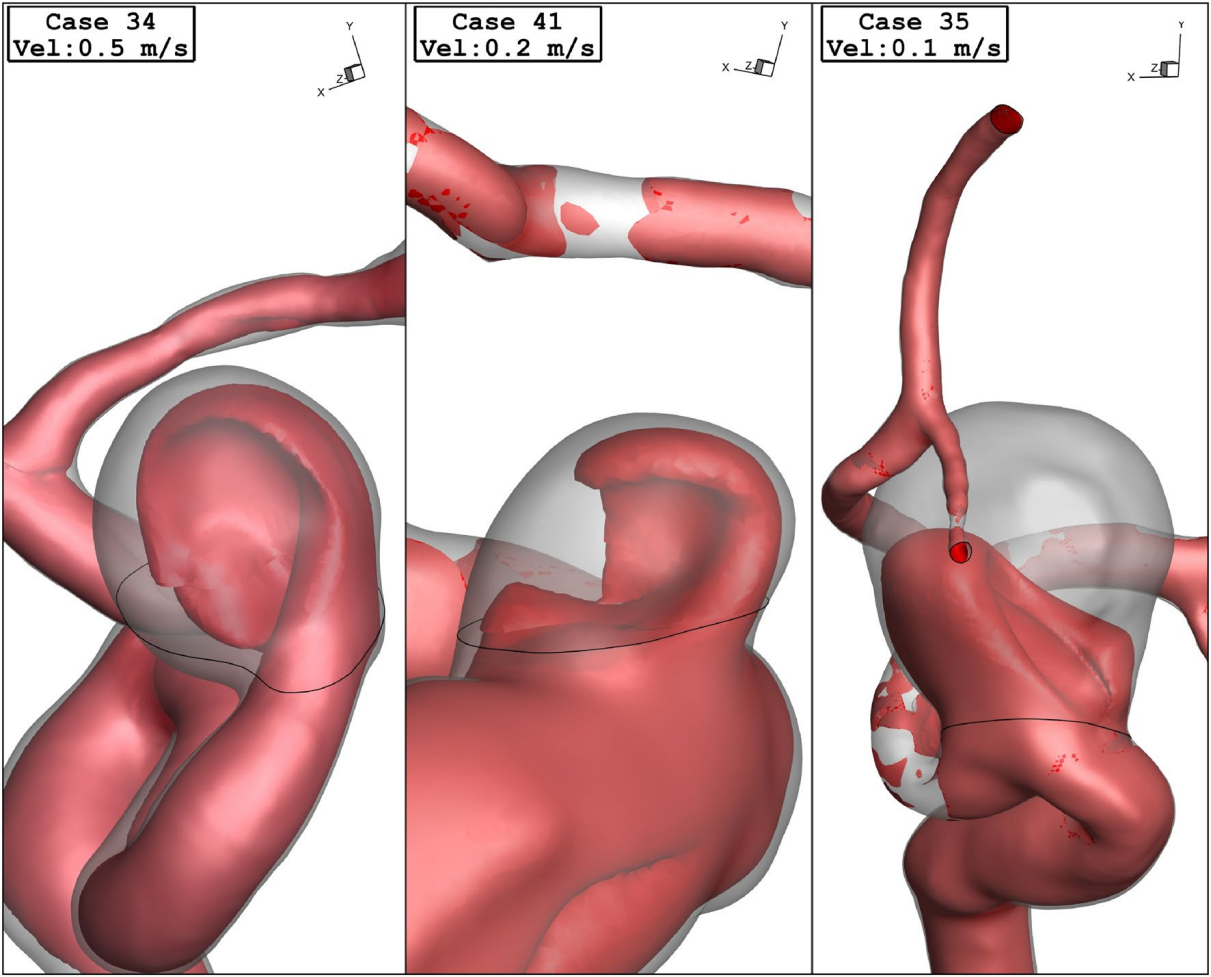
Iso-Surface (velocity at peak systolic) in different cases.

Figure 10 displays the effects of the parent vessel's mean diameter on the mean sac velocity inside the sac domain of the chosen cases. For case 34, the changes in the mean sac velocity have no meaningful connection to the parent vessel's mean diameter. Figure 11 demonstrates the streamline pattern inside the aneurysms and streamlines are colored by the velocity magnitude at peak systolic. The streamline indicates that the velocity of the blood inside the sac domain is lower than the main parent vessel. To demonstrate the blood flow structure, Fig. 12 displays the iso-velocity of the blood in the sac section which is filled by the coiling porosity. The blood velocity feature indicates the main shape of flow when a fraction of the domain is filled by the coil.

## Conclusion

The present study investigates the influence of sac section area and parent vessel diameter on the hemodynamic feature of the blood flow in selected aneurysms. Computational fluid dynamic is applied for the modeling of the transient blood stream in the cardiac cycle. The selected aneurysms are treated and filled by the porous media. The variation of the mean OSI shows that increasing the diameter of the parent vessel directly decreases the OSI value on the sac surface. In addition, the mean WSS decreases with the increase of the parent vessel diameter.

## Data Availability

All data generated or analysed during this study are included in this published article.
